# Post-activation performance enhancement (PAPE) protocols do not further increase jumping performance beyond warm-up effects: findings from three acute randomized crossover trials

**DOI:** 10.3389/fphys.2024.1447421

**Published:** 2024-08-14

**Authors:** Ludwig Rappelt, Steffen Held, Tim Wiedenmann, Florian Micke, Lars Donath

**Affiliations:** ^1^ Department of Intervention Research in Exercise Training, German Sport University Cologne, Cologne, Germany; ^2^ Department of Movement and Training Science, University of Wuppertal, Wuppertal, Germany; ^3^ Department of Sport and Management, IST University of Applied Sciences, Düsseldorf, Germany; ^4^ Department of Sports Science, Bielefeld University, Bielefeld, Germany

**Keywords:** PAP, counter-movement-jump, potentiation, resistance training, maximal voluntary contraction, muscle, power

## Abstract

**Introduction:** Post-activation performance enhancement (PAPE) cannot be clearly distinguished from and may be explained in large by warm-up effects. To disentangle PAPE from a systemic warm-up effect, we conducted three randomized crossover trials (RCT).

**Methods:** Each RCT consisted of a familiarization/one-repetition-maximum (1RM) assessment session followed by two interventional sessions (random order). In Study I, 18 participants (age: 26 ± 4 years; height: 1.84 ± 0.06 m; mass: 83.7 ± 8.7 kg; Squat-1RM: 146 ± 19 kg) performed either a 3-s isometric squat at 130%1RM or a 6-s isometric squat at 65%1RM. In Study II, 28 participants (11 female; age: 23 ± 3 years; height: 1.77 ± 0.08 m; mass: 76.5 ± 10.4 kg; Squat-1RM: 109 ± 38 kg) completed either Squat (3 × 3 repetitions, 85%1RM) or local electromyostimulation of the quadriceps muscle (85% of individual pain threshold). In Study III, 20 participants (6 female, age: 25.0 ± 3.5 years, mass: 78.5 ± 15.8 kg, height: 1.75 ± 0.08 m; SQ-1RM: 114 ± 33 kg, chest-press-1RM: 74 ± 29 kg) performed either squats or chest press (4 repetitions, 80%1RM). Counter-Movement-Jump height (CMJ) was assessed after a general (PRE) and/or muscle-specific warm-up (POST_WU) and for up to 11 min after the PAPE protocols. To identify possible differences in CMJ between the experimental conditions, mixed-design ANOVA models were used for each study individually, with condition and time modelled as fixed effects, while participants were included as a random effect blocking factor. The level of statistical significance was set at α = 5%.

**Results:** In studies I and II, significant effects for time (*p* < 0.05, ω_p_^2^ = 0.06 and *p* < 0.001, ω_p_^2^ = 0.43) were found with the highest CMJ compared to all other time points at PRE (≤8.2 ± 4.6%, standardized mean difference: ≤0.39), regardless of condition. In study III, no significant effects were observed.

**Discussion:** Thus, PAPE protocols do not further improve jumping performance compared to a general and muscle-specific traditional warm-up. Prior to tasks requiring explosive strength, general and sport-specific warm-up strategies should be used.

## 1 Introduction

Since the mid-to-late 1800s, terms like “staircase,” “post-tetanic potentiation,” and “post-activation potentiation” (PAP) have been used to describe the phenomenon of increased muscle contractile force following prior conditioning contractions, typically involving stimulation of the muscle or its motor nerve (Blazevich and Babault, 2019). In this context, PAP traditionally refers to the well-described phenomenon in which a conditioning contraction (such as a near-maximal voluntary contraction (MVC) or an electrically induced tetanic contraction lasting less than 10 s) leads to enhanced twitch tension and rate of tension development, with a half-life of approximately 28 s (Vandervoort et al., 1983). Additionally, this conditioning contraction results in reduced post-stimulus relaxation time compared to the baseline twitch evoked in a resting muscle prior to the contraction (Sale, 2002; Blazevich and Babault, 2019).

Subsequently, the term PAP was however broadened to encompass a temporal increase in performance metrics such as vertical jump height, sprinting, or ballistic movements reaching their peak at 3–7 min (Wilson et al., 2013), 5–7 min (Seitz and Haff, 2016), >8 min (Gouvêa et al., 2013; Seitz and Haff, 2016), 7–8 min (Garbisu-Hualde and Santos-Concejero, 2021), or 7–10 min (Lesinski et al., 2013; Wilson et al., 2013) after a moderate-to-high-load strengthening exercise (Prieske et al., 2020). Some studies even reported potentiation effects, as measured through vertical jump performance, following a full training session that included both strength training and technical elements in professional volleyballers (Berriel et al., 2022), or in runners after completing a 30 km self-paced time trial (Rosso et al., 2016). Consequently, to differentiate between enhancements via electrically evoked contractions (as in PAP) and the enhancement of voluntary movements, it has been recommended to use the term post-activation performance enhancement (PAPE) for the latter (Cuenca-Fernández et al., 2017; Blazevich and Babault, 2019; Prieske et al., 2020).

The primary mechanism underlying PAP, namely, the phosphorylation of myosin regulatory light chains, leading to enhanced sensitivity of actin-myosin interaction to calcium ions released from the sarcoplasmic reticulum (Sweeney et al., 1993; Rassier and Macintosh, 2000), is well-described. The mechanisms underlying PAPE, however, unreliable in its occurrence and questionable in its magnitude (Dobbs et al., 2019; Prieske et al., 2020), remain somewhat unclear. Possible physiological mechanisms accounting for PAPE may involve several factors: (I) an increase in muscle temperature, which positively influences the temperature-sensitive myosin ATPase reaction; (II) elevated blood flow, leading to a shift of water into the intracellular space, potentially decreasing Ca^2+^ sensitivity; and (III) increases in spinal-level excitability, associated with changes in arousal level and enhancements in tendon tap and H-reflex amplitudes (Blazevich and Babault, 2019). However, these mechanisms are almost entirely consistent with the effects that are also thought to be responsible for the effects of a warm-up routine before exercise (e.g., increased muscle temperature, elevated blood flow to the muscles and increased nerve conduction rate) (Bishop, 2003a). While the definitive ideal intensity of a warm-up is not clearly defined, the consensus seems to be a mild sweat without fatigue (Safran et al., 1989). In this context, traditionally, a warm-up is thought to consist of general and sport-specific (e.g., specific stretches and sport-related movements) parts comprising light aerobic activities (e.g., jogging) and resistive exercises (Kulund and Töttössy, 1983; Safran et al., 1989). For example, a significantly higher jumping performance was reported, when including five loaded jumps (10% of bodyweight as additional load) or a series of submaximal (5 × 2 repetitions at 20% of the one-repetition-maximum (1RM) to 90%1RM) half-squats (Gourgoulis et al., 2003) into the warm-up routine. A sufficient warm-up protocol will thus subsequently increase performance metrics such as vertical jump height (Burkett et al., 2005), sprinting performance (Grodjinovsky and Magel, 1970), or power-related tasks (Sargeant and Dolan, 1987) – generally 3–5 min after the warm-up protocol (Bishop, 2003b), thus showing a close temporal relationship with the peak in performance after administering a PAPE protocol.

Even though the conditioning activity to induce a PAPE effect is thought to require a carefully balanced combination of intensity (Gołaś et al., 2016), volume and recovery time (Kilduff et al., 2008), meta-analyses are unequivocal and inconclusive in their findings for optimal protocols. Partially cofounded by athletes’ level of performance, trivial to moderate (Gouvêa et al., 2013; Wilson et al., 2013; Seitz and Haff, 2016; Dobbs et al., 2019; Krzysztofik et al., 2020) PAPE effects have been reported 3–10 min (Gouvêa et al., 2013; Lesinski et al., 2013; Wilson et al., 2013; Seitz and Haff, 2016; Garbisu-Hualde and Santos-Concejero, 2021) after moderate to high intensity preconditioning activities (60%1RM to >90%1RM) performed in a single set (Krzysztofik et al., 2020), multiple sets (Lesinski et al., 2013; Wilson et al., 2013; Seitz and Haff, 2016), or regardless of the number of sets (Dobbs et al., 2019; Garbisu-Hualde and Santos-Concejero, 2021). Thus, as there is no clear distinction between PAPE protocols and (normal) warm-up protocols, it is reasonable to assume, that the effect of PAPE protocols may be interfered with or attributed to local or even systemic warm-up effects (Macintosh et al., 2012; Blazevich and Babault, 2019; Prieske et al., 2020). This notion is supported by studies reporting a PAPE effect in the upper body after performing a conditioning activity in the lower body (Caldeira et al., 2023) or *vice versa* (Bartolomei et al., 2023). Moreover, studies reporting large PAPE effects often implemented only a very limited warm-up [e.g., 5 min of light cycling and/or dynamic stretching (Lowery et al., 2012; Esformes and Bampouras, 2013)] before obtaining baseline values of their outcome measure. However, it has been emphasized that ≤5 min of low-intensity exercise as warm-up does not yield significant short-term performance enhancements (Bishop, 2003b). Therefore, it remains open, whether an additional PAPE effect will be visible when performing a sufficient warm-up routine consisting of both general and specific exercises at moderate intensities prior to the conditioning activity.

Against this background, we conducted three acute randomized crossover trials comprising different PAPE protocols after a warm-up routine consisting of general and specific exercises. Each trial incorporated a different PAPE conditioning activity, while being orientated on the aforementioned recommendations in terms of intensity, volume and recovery intervals. The first study focused on isometric contractions as a conditioning activity. When performing maximal voluntary isometric contractions, studies have reported lower PAPE effects (Tillin and Bishop, 2009; Dobbs et al., 2019). However, it still remains unclear whether these lower effects result from fatiguing effects due to an inadequate balance of load and recovery time (Kilduff et al., 2008) or a lack of movement similarity (Dello Iacono et al., 2018). Thus, in our first study, we aimed at investigating whether performing isometric contractions at submaximal intensities and with shorter time-under-tension may be sufficient to induce further performance improvements (i.e., PAPE effects) after an adequate warm-up routine. Secondly, exploring alternative activation protocols, neither local electrical stimulation of the quadriceps muscle (Sari et al., 2022), nor whole-body electromyostimulation (EMS) (Dote-Montero et al., 2021) showed significant performance increases. Notably, however, both studies used electrical stimulation at a frequency of 100 Hz. Considering that a higher percentage of type-II muscle fibers is associated with increased PAPE effects (Hodgson et al., 2005; Blazevich and Babault, 2019; Garbisu-Hualde and Santos-Concejero, 2021), and that type-II fibers can be recruited via EMS at (very) low stimulation frequencies and intensities (Maffiuletti et al., 2006), it appears reasonable to expect that applying electrical stimulation at lower frequencies could induce PAPE effects without the need for additional high loads. Therefore, in our second randomized crossover trial, we tested the hypothesis that the application of local electrical stimulation of the quadriceps muscle results in a lower PAPE effect than a traditional PAPE protocol consisting of squats as a conditioning activity after an adequate warm-up routine. Finally, in the third trial we aimed at elucidating whether a PAPE protocol for the upper body may result in a lower PAPE effect than a traditional PAPE protocol consisting of squats. All three randomized controlled trials focused on Counter-Movement-Jump performance as the primary (and sole) outcome measure. These results may influence the use of PAPE protocols as part of a warm-up routine to acutely improve strength-related performance in trained participants.

## 2 Material and methods

### 2.1 Study I: sub-vs. supramaximal isometric Squat–participants and study design

Based on the effect size reported in previous research utilizing isometric leg press as an activation condition (Tsolakis et al., 2011), a power analysis using G*Power (Version 3.1.9.6, *α* = 0.05, study power (1-*β*-error) = 0.80, r = 0.5, effect size η_p_^2^ = 0.11 (*f* = 0.352)) revealed a required sample size of n = 16. Based on our previous experience of acute intervention trials (Held et al., 2021; Rappelt et al., 2021), we assumed only low dropouts. Thus, a convenience sample of n = 18 healthy young male participants (age: 26 ± 4 years; height: 1.84 ± 0.06 m; mass: 83.7 ± 8.7 kg; Squat one-repetition-maximum (1RM): 146 ± 19 kg) was enrolled in this acute randomized controlled crossover trial. Inclusion criteria were (I) aged ≥18 years, (II) at least 2 years of experience in strength training, (III) Squat-1RM of at least 125% of body mass (IV) no acute or chronic medical condition that potentially impede the completion of all experimental sessions. Based on the classification framework suggested by McKay and colleagues, participants may be classified as “Trained” (Tier 2) (McKay et al., 2022). The study protocol was approved by the local ethical committee (193/2022), and all participants provided informed written consent before the start of the study.

The study design comprised three lab visits over a 2-week period as follows: (I) anthropometric evaluations, determination of Squat-1RM, and Counter-Movement-Jump (CMJ) familiarization (II and III, randomized order determined by coin tossing) PAPE protocol with isometric squatting at 130% of 1RM (ISO-PAPE-130) or isometric squatting at 65% of 1RM (ISO-PAPE-65); CMJ evaluation after a general warm-up (PRE; 5 min of cycling), as well as 2 (POST_2), and 6 min (POST_6) after the respective PAPE protocols (see testing procedure for details). All laboratory visits were spaced at least 48 h, but at maximum 1 week apart, consistently scheduled at the same time of day to minimize circadian influences.

### 2.2 Study I. Sub-vs. supramaximal isometric Squat–Testing procedures

To establish the 1RM for the half-squat, participants underwent a structured protocol during the initial familiarization session: In short, following 5 min of cycling (resistance set at 150% of body mass in W at 80 rpm), participants performed submaximal warm-up sets comprising three repetitions at 30%, 40%, 50%, 60% and 70% of their estimated 1RM, respectively with 3 min of rest between sets. Subsequently, participants executed two repetitions at 80% and a single repetition at 90% of their estimated 1RM. Thereafter, to determine their actual 1RM, participants were instructed to perform the concentric phase of the squat at maximal velocity. For this, an additional load equivalent to 95% and 100% of their estimated 1RM was used. The mean propulsive velocity (MPV) of the bar was recorded using a linear position transducer (Vitruve encoder, Vitruve, Madrid, Spain) vertically attached to the barbell using a Velcro strap. This device has been validated and showed excellent reliability for recording the MPV (Standard error of the measurement (SEM) < 0.01 m·s^−1^; coefficient of variation (CV) < 1.8%; intra-lass correlation coefficient (ICC) > 0.999) (Martínez-Cava et al., 2020). Data were recorded at a sampling rate of 1,000 Hz, and MPV was subsequently computed by differentiating the transducer displacement data with respect to time. The sensor was connected via Wi-Fi to a smartphone (iPhone 10, Apple, Silicon Valley, CA, United States) to enable simultaneous display of MPV using the Vitruve App. As the expected MPV at the 1RM for the half-squat is 0.30 m·s^-1^ (Loturco et al., 2016), the additional load was gradually increased by 2.5%–5.0% above the estimated 1RM until MPV dropped below 0.30 m·s^-1^. To mitigate fatigue, participants achieved their 1RM (defined as the lowest load at which MPV <0.30 m·s^-1^) during the third or fourth attempt at the latest, with 4 min of rest granted between attempts.

In both ISO-PAPE-65 and ISO-PAPE-130, participants began with a general warm-up of 5 min of cycling (resistance in Watt set at 150% of body mass at a cadence of 80 rpm) followed by half-squats. They performed five repetitions at 30%1RM, four repetitions at 50%1RM, three repetitions at 70%1RM and one repetition at 90%1RM (inner knee-angle between 180° and 110°). For the subsequent PAPE conditioning protocol, participants were instructed to position themselves in a steady stand with knees slightly flexed to allow for a pre-activation of the lower extremities and to maintain this posture. The study administrators, consisting of two individuals, loaded participants with a barbell at either 65% (ISO-PAPE-65) or 130% (ISO-PAPE-130) of their 1RM. Participants then performed either a 3-s isometric squat (ISO-PAPE-130) or a 6-s isometric squat (ISO-PAPE-65). Thus, both protocols were matched in terms of absolute workload (repetitions × load × time under tension).

After the general warm-up (PRE), as well as at 2 min (POST_2) and 6 min (POST_6) following the respective PAPE protocol, CMJ performance was evaluated using a force plate recording ground reaction force at 1,000 Hz (FP4060-15 - TM-4000, Bertec Corporation, Columbus, United States). Participants were instructed to keep their arms placed on their hips (akimbo). Participants performed three trials of CMJ with approximately 15 s of rest between attempts. Jump height was determined using the flight time method and calculated as jumping height [in m] = 9.81 × flight time [in s]^2^/8 (= flight time [in s]^2^ × 1.22625) (Bosco et al., 1983). For this, take-off and landing were identified using a 10 N threshold. Previous studies have reported high reliability for jumping height assessment via force plate (ICC = 0.92–0.98, CV = 1.3–4.1) (Hori et al., 2009). The respective two best trials of for each time point were averaged and used for all further analyses.

### 2.3 Study II. Electrical stimulation vs. Squat–participants and study design

Assuming low effects based on previously published research using Squats in a PAPE activation protocol with a similar design (Fukutani et al., 2014; Bauer et al., 2019), a power analysis using G*Power (*α* = 0.05, study power (1-*β*-error) = 0.80, r = 0.7, effect size η_p_^2^ = 0.03 (f = 0.176)) revealed a required sample size of n = 26. Again, assuming low dropouts, a convenience sample of n = 28 young healthy participants (11 female; age: 23 ± 3 years; height: 1.77 ± 0.08 m; mass: 76.5 ± 10.4 kg; Squat-1RM: 109 ± 38 kg) were enrolled in this acute randomized controlled crossover trial. Again, participants can be classified as “Trained” (Tier 2). Inclusion criteria were (I) aged ≥18 years, (II) at least 2 years of experience in strength training, (III) actively pursuing a sport involving regular jumping movements (e.g., soccer, handball, volleyball) and (IV) having no acute or chronic medical condition that potentially impede the completion of all experimental sessions. The study protocol received approval from the local ethical committee (042/2024), and all participants provided informed written consent before the study commencement.

The study comprised three laboratory visits over a 2-week period: (I) Anthropometric assessments; determination of Squat-1RM; identification and marking of motor muscle points on the vastus lateralis and vastus medialis for electrode placement; determination of EMS intensity at the individual pain threshold (iPT); familiarization with EMS through five cycles of increasing stimulation intensity starting from 1 mA; and familiarization with CMJ. (II and III in randomized order as determined by coin toss) PAPE protocol administered via superimposed EMS (EMS-PAPE) or via squatting exercise (SQ-PAPE) with CMJ evaluations conducted after a general (PRE), immediately following a protocol-specific warm-up (POST_WU), as well as 3 min (POST_3), 7 min (POST_7) and 11 min (POST_11) after the respective PAPE interventions (see testing procedure). All laboratory visits were spaced at least 48 h, but at maximum 1 week apart, consistently scheduled at the same time of day to minimize circadian influences.

### 2.4 Study II. Electrical stimulation vs. Squat–testing procedures

For EMS-PAPE, using a commercially available EMS device (Compex SP 8.0; Medicompex SA, Ecublens, Switzerland), electrical stimulation was delivered bilaterally to the m. vastus lateralis and the m. vastus medialis via electrical cords. A single rectangular negative electrode (length × height: 10 cm × 5 cm) was placed to the right of and just under the femoral triangle, while two square positive electrodes (5 cm × 5 cm) were positioned near the proximal insertion of the respective muscles of both legs. To standardize electrode placement between participants, the respective muscle motor points [i.e., the location on the surface of the skin above a muscle at which a transcutaneous applied electrical impulse with the least injected current evokes a muscle twitch (Gobbo et al., 2014)] were detected during the familiarization session using a motor point pen (Compex Motor Point Pen; Medicompex SA, Ecublens, Switzerland). For this, a dispersive electrode was placed over the antagonist, and gel was applied to the skin to reduce resistance and facilitate the flow of electric current. Starting at a low stimulation frequency of 5 Hz and a stimulation intensity of 1 mA, the negative pen electrode was gradually moved over the belly of the respective muscle to evoke muscle twitching. If no muscle twitch was visually detectable, the stimulation intensity was incrementally increased (Gobbo et al., 2014). The motor point’s location was then marked on the skin’s surface with waterproof ink visible for the duration of the study to allow for exact replication of the electrode positioning between session. During the familiarization session, the electrical stimulation intensity, at which the perceived pain could just be tolerated (individual maximal tolerable pain threshold; iPT) was determined. For this purpose, starting at 1 mA (stimulation frequency: 70 Hz) the intensity was continuously increased by 0.25 mA until participants could no longer tolerate the induced pain. The maximal stimulation intensity that could be endured (in mA) was subsequently defined as 100%iPT. Due to individual differences in tissue structure resistance, it is not possible to precisely determine the intensity ultimately reaching the muscles (Lake, 1992).

During the EMS-PAPE condition, following the general warm-up (5 min of cycling with resistance set at 150% of body mass in W at 80 rpm), participants engaged in a specific warm-up consisting of two sets of 3 × 10 s of isometric squatting with complementary electrical stimulation. In both sets, participants were instructed to take a squatting position with their thighs parallel to the floor and maintain this position for 10 s. During this time, electrical stimulation at a frequency of 70 Hz and an intensity (in mA) of 45% and 65%iPT for the first and second set, respectively, was administered. Subsequently, after holding the position for 10 s, participants returned to an upright position for a brief rest period (5 s with 9 Hz of superimposed electric stimulation). For the subsequent PAPE conditioning activity, participants performed three sets of 3 × 10 s of isometric squatting with complementary electrical stimulation (70 Hz at 85%iPT), interspersed with 5 s of upright standing (9 Hz). The resting period between all sets was set at 2 min.

For SQ-PAPE, following the general warm-up (5 min of cycling with resistance set at 150% of body mass in W at 80 rpm), participants underwent a specific warm-up involving squatting (2 sets of 3 repetitions, with a 1.5-s eccentric and concentric movement). Participants were instructed to reach a squatting position with their thighs parallel to the floor and then return to an upright position. Additional loads of 45% and 65% of their 1RM were applied for the first and second warm-up sets, respectively. During the PAPE conditioning phase of SQ-PAPE, participants completed 3 × 3 repetitions at 85%1RM, with 1.5 s of eccentric and concentric movement, respectively. The resting period between all sets was set at 2 min. To estimate the 1RM, participants followed a structured protocol (Baechle et al., 2000) during the familiarization session. In short, this protocol involved three warm-up sets with self-selected submaximal loads, followed by repetitions with a submaximal load aiming for failure within a range of 1–10 repetitions maximum (1-10RM). Based on the maximum number of repetitions and the respective load, the 1RM could then be estimated using the equation provided by Baechle and colleagues (Baechle et al., 2000) (load [in kg] × 0.033 × repetitions).

After the general (PRE) and specific warm-up (POST_WU), as well as 3 min (POST_3), 7 min (POST_7), and 11 min (POST_11) following the respective PAPE protocol, CMJ performance was evaluated using the same procedure as described earlier.

### 2.5 Study III: upper vs. lower body–participants and study design

Based on the findings of study II, a power analysis using G*Power [Version 3.1.9.6, *α* = 0.05, study power (1-*β*-error) = 0.80, r = 0.944, effect size η_p_^2^ = 0.009 (f = 0.095)] indicated a required sample size of n = 16. Again, assuming only low dropouts, a convenience sample of n = 20 participants (6 female, age: 25.0 ± 3.5 years, mass: 78.5 ± 15.8 kg, height: 1.75 ± 0.08 m; SQ-1RM: 114 ± 33 kg, chest-press-1RM: 74 ± 29 kg) was recruited for this acute randomized controlled crossover trial. Again, participants can be classified as “Trained” (Tier 2). Inclusion criteria were (I) being aged ≥18 years, (II) possessing at least 2 years of experience in strength training, and (III) having no acute or chronic medical conditions that could impede the completion of all experimental sessions. This study protocol was approved by the local ethical committee (208/2023), and all participants signed an informed written consent prior to the start of the study.

Like studies I and II, this third study also involved three laboratory visits over a 2-week period, structured as follows: (I) Anthropometric assessments; determination of Squat-1RM and Chest-Press-1RM; CMJ familiarization. (II and III in randomized order, decided per coin toss) PAPE protocol on either the Chest-Press machine (CP-PAPE) or through squatting exercise (SQ-PAPE), with CMJ evaluations conducted after a general (PRE) and protocol-specific (POST_WU) warm up, as well as 3 min (POST_3), 7 min (POST_7), and 11 min (POST_11) following the respective PAPE protocols (see testing procedure for a detailed description). All laboratory visits were spaced at least 48 h, but at maximum 1 week apart, consistently scheduled at the same time of day to minimize circadian influences.

### 2.6 Study III: upper vs. lower body–testing procedures

CP-PAPE was performed at a seated chest press machine (Edition-Line, gym80, Gelsenkirchen, Germany). Participants sat upright with a hip angle of 90°, ensuring back support, and kept their feet firmly planted on the ground. In this position, seat height was adjusted to align the machine’s handlebars with the participants’ sternum. Starting with extended elbows and hands positioned at chest level (wrist in a neutral position), participants were instructed to push forward until their arms reached full extension. During the SQ-PAPE protocol, participants performed half-squats with additional load using an Olympic long bar. They were instructed to lower themselves until their thighs were parallel to the floor and then return to an upright position (i.e., similar to the movement performed in study II). Movement speed was standardized using a metronome set at a consistent pace of 1.5 s for both the lowering and lifting phases.

In both conditions, participants started with a general warm-up comprising 5 min of cycling with a resistance set at 150% of body mass in W at 80 rpm. For the protocol-specific warm-up, participants executed 10 repetitions (SQ-PAPE: bar only; CP-PAPE: 10 kg), followed by six repetitions at 40%1RM, four repetitions at 60%1RM and two repetitions at 70%1RM, either on the chest press (during CP-PAPE) or with squats (SQ-PAPE). For the subsequent PAPE conditioning, participants performed four repetitions at 80% 1RM. To determine the 1RM for the squats and the chest press, participants followed a structured protocol (Baechle et al., 2000) during the familiarization session. In brief, after a submaximal warm-up set consisting of 10 repetitions (SQ-PAPE: bar only; CP-PAPE: 10 kg) as well as 8, 4, 2, and 1 repetition at 50%, 60%, 80%, and 90% of the estimated 1RM, respectively, the load was gradually increased (0.5–5.0 kg). This procedure continued until the 1RM, or failure was reached. To prevent fatigue, it was ensured that participants reached their 1RM during the third or fourth attempt at the latest.

After the general (PRE) and specific warm-up (POST_WU), as well as 3 min (POST_3), 7 min (POST_7), and 11 min (POST_11) following the respective PAPE protocol, CMJ performance was evaluated using the same procedure as described earlier.

### 2.7 Statistics

If not stated otherwise, all data are presented as mean ± standard deviation (SD). Normal distribution was verified via the Shapiro–Wilk test (*p* ≥ 0.1) and investigation of residuals using Q–Q plots. Variance homogeneity was verified employing Levene-tests (*p* ≥ 0.1). Furthermore, no outliers (≥Q3 + 1.5 × interquartile range [IQR = Q3 - Q1] or ≤ Q1 - 1.5 × IQR) were found in the raw data. To assess the consistency of all three jumps per time point within each subject, Intraclass Correlation Coefficients (ICC) were calculated [two-way mixed-effects model for consistency; ICC(3,1)]. ICCs were rated as excellent (0.9–1), good (0.74–0.9), moderate (0.4–0.73) and poor (0–0.39). Moreover, to allow for a better interpretation of differences in jumping height between time points, the mean difference between the two highest jumps at PRE and the respective limits of agreement (LoA: 1.96 × SD of the difference between jumps) were analysed. To identify possible differences in jumping height between the two experimental conditions and the three (study I) or five time points (study II and III), a 2 (condition: ISO-PAPE-130 vs. ISO-PAPE-65) × 3 (time: PRE, POST_2 and POST_6), respective, 2 (condition: SQ-PAPE vs. EMS-PAPE (study II) or CP-PAPE (study III)) × 5 (time: PRE, POST_WU, POST_3, POST_7, and POST_11) experimental design was used. *Condition* and *time* were modelled as fixed effects, while participants were included as a random effect blocking factor. Fixed effects were analysed using F-tests (type III) with Satterthwaite approximations for the degrees of freedom. ANOVA effect sizes are given as partial omega squared (ω_p_^2^), with ≥0.01, ≥0.06, ≥0.14 indicating small, moderate, and large effects, respectively (Cohen, 1988). Subsequently, in case of a statistically significant interaction effect or main effect of time, Tukey *post hoc* tests to adjust for multiple testing were computed. For pairwise effect size comparison, standardized mean differences (SMD) were calculated as differences between means divided by the pooled standard deviations (trivial: | SMD | < 0.2, small: 0.2 ≤ | SMD | < 0.5, moderate: 0.5 ≤ | SMD | < 0.8, large: | SMD | ≥ 0.8) (Cohen, 1988). All statistical analyses were performed using *R* (version 4.2.0) in its integrated development environment *RStudio* (version 2023.06.1 + 524). The ICC analysis was conducted using the *ICC()*-function from the *psych* package (Revelle, 2024). Mean differences and LoA were calculated via the *bland. altman.stats()*-function of the package *BlandAltmanLeh* (Lehnert, 2015). The package *rstatix* (Kassambara, 2023) was used for analysis of outliers and performing Shapiro–Wilk tests. For Levene-tests the *car* package (Fox et al., 2023) was used. Mixed modelling was performed using the *lmerTest* package (Kuznetsova et al., 2017) and the *anova()*-wrapper of *stats* (part of base *R*) was used to provide ANOVA tables. Effect size estimation was performed using the *effectsize* package (Ben-Shachar et al., 2020). Post-hoc testing (Tukey) was performed via the *emmeans* package (Lenth et al., 2022). For all statistical analyses, a *p*-value below 0.05 was considered as statistically significant. All relevant data supporting the conclusion of this article is available under the following link: https://osf.io/hj9ux/. The MD5 hash of the Excel-Spreadsheet containing all data (“CMJ_data.xlsx”) is *ce02387b9df65900bddf4051a3b6ea27*.

## 3 Results

### 3.1 Agreement and mean difference

The calculated ICC(3,1) point estimate for all three trials can be considered as excellent (Study I: 0.954, 95% confidence interval (CI) [0.922; 0.975]; Study 2: 0.968, 95%CI [0.951; 0.980]; Study III: 0.938, 95%CI [0.899; 0.965]). Mean difference between the respective two highest jumps at PRE were −0.009 m [LoA: 0.025–0.007 m] for study I, −0.011 m [-0.034–0.012 m] for study II, and −0.012 m [-0.037–0.013 m] for study III.

### 3.2 Study I: sub-vs. supramaximal isometric Squat

**FIGURE 1:**
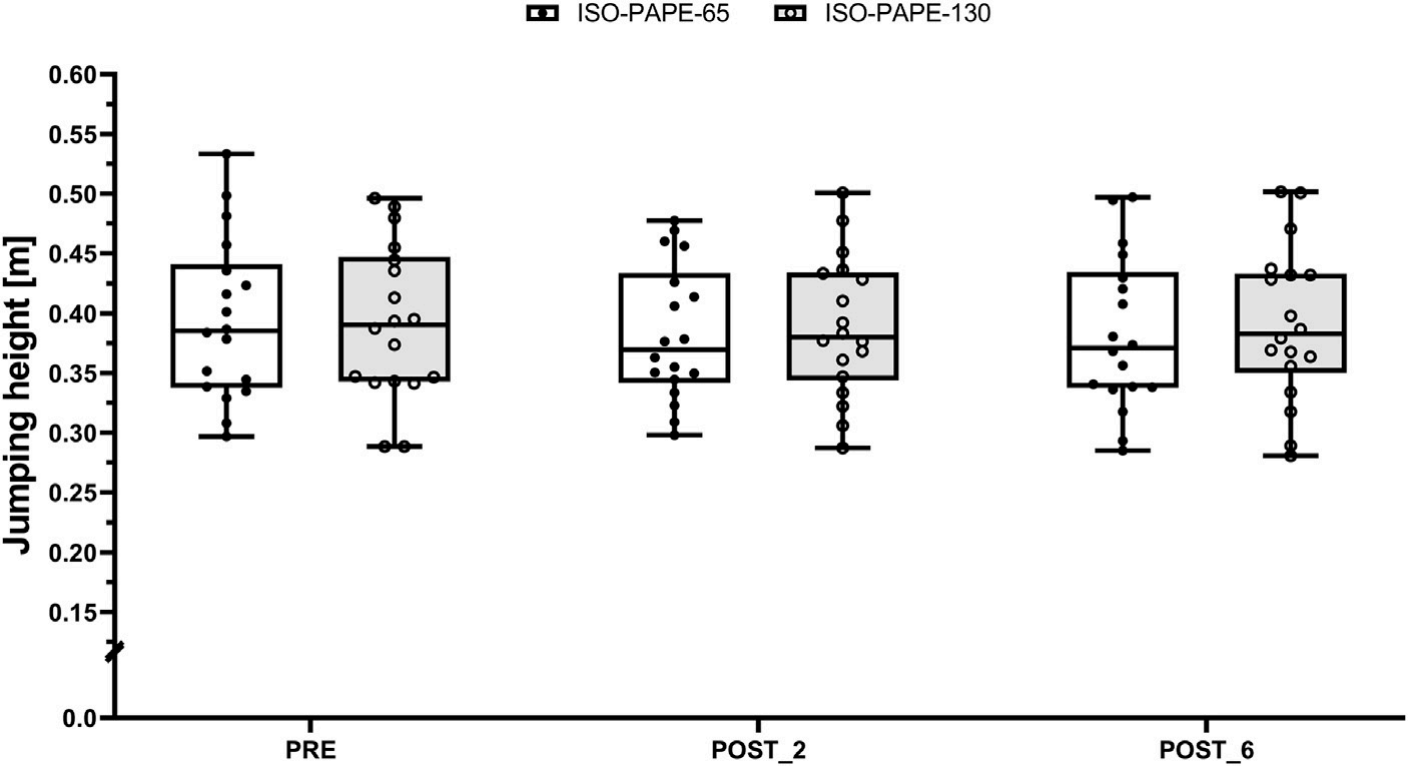
Boxplot (Q1 to Q3, including Median), and Whiskers (showing minimum and maximum values) for Counter-Movement-Jumping height after the general warm-up (PRE) as well as 2 (POST_2) and 6 min (POST_6) after the respective PAPE protocols with an additional load of 65% of one repetition maximum (ISO-PAPE-65; white box) and 130% of one repetition maximum (ISO-PAPE-130; grey box). Individual values are also depicted (black circles for ISO-PAPE-65 and white circles for ISO-PAPE-130).

CMJ neither exhibited a statistically significant interaction effect (F (2, 85) = 1.74, *p* = 0.182, ω_p_^2^ = 0.02 [small effect size]) nor a statistically significant main effect for condition (F (1, 85) = 2.73, *p* = 0.102, ω_p_^2^ = 0.02 [small effect size]). However, a statistically significant effect for time was observed (F (2, 85) = 3.67, *p* < 0.05, ω_p_^2^ = 0.06 [moderate effect size]). Subsequent *post hoc* analysis revealed statistically significant differences between PRE and POST_2 (t (85) = −0.43, *p* = 0.035, 95%CI [-0.015, −0.0004], SMD = 0.06 [trivial effect size])). Consequently, CMJ jumping height slightly decreased from PRE to POST_2, regardless of the condition (ISO-PAPE-130: 0.392 ± 0.064 m to 0.388 ± 0.059 m, SMD = 0.03 [trivial effect size] and ISO-PAPE-65: 0.394 ± 0.067 m to 0.383 ± 0.057 m, SMD = 0.09 [trivial effect size]) (Figure 1).

### 3.3 Study II: electrical stimulation vs. Squat

**FIGURE 2:**
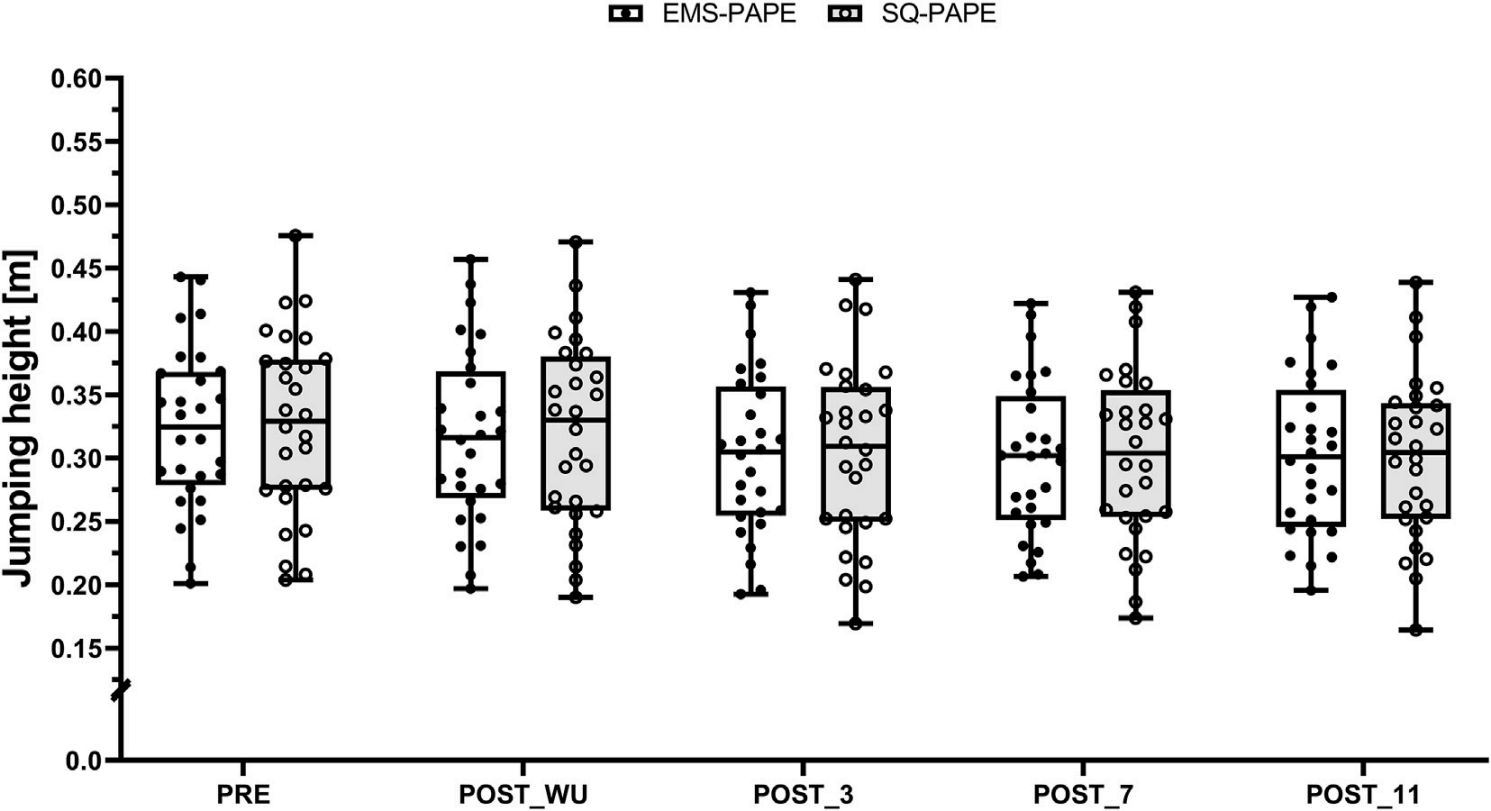
Boxplot (Q1 to Q3, including Median), and Whiskers (showing minimum and maximum values) for Counter-Movement-Jumping height after the general (PRE) and the specific warm-up (POST_WU) as well as 3 (POST_3), 7 (POST_7), and 11 min (POST_11) after the respective PAPE protocols performed with electrical stimulation (EMS-PAPE; white box) and squats (SQ-PAPE; grey box). Individual values are also depicted (black circles for EMS-PAPE and white circles for SQ-PAPE).

CMJ neither revealed a statistically significant interaction effect (F (4, 243) = 0.49, *p* = 0.746, ω_p_^2^ < 0.001 [trivial effect size]) nor a statistically significant main effect for condition (F (1, 243) = 1.55, *p* = 0.214, ω_p_^2^ = 0.002 [trivial effect size]). However, a statistically significant main effect for time was observed (F (4, 243) = 47.8, *p* < 0.001, ω_p_^2^ = 0.43 [large effect size]). Subsequent *post hoc* analysis revealed significant differences between PRE and POST_WU (t (243) = 3.32, *p* = 0.009, 95%CI [0.001, 0.014], SMD = 0.05 [trivial effect size])), POST_3 (t (243) = 9.44, *p* < 0.001, 95%CI [0.015, 0.028], SMD = 0.16 [trivial effect size]), POST_7 (t (243) = 10.88, *p* < 0.001, 95%CI [0.019, 0.031], SMD = 0.18 [trivial effect size]), and POST_11 (t (243) = 10.51, *p* < 0.001, 95%CI [0.018, 0.030], SMD = 0.18 [trivial effect size]). Furthermore, statistically significant differences were found between POST_WU and POST_3 (t (243) = 6.12, *p* < 0.001, 95%CI [0.008, 0.020], SMD = 0.10 [trivial effect size]), POST_7 (t (243) = 7.56, *p* < 0.001, 95%CI [0.011, 0.024], SMD = 0.12 [trivial effect size]), and POST_11 (t (243) = 7.19, *p* < 0.001, 95%CI [0.010, 0.023], SMD = 0.12 [trivial effect size]) (Figure 2). Consequently, the CMJ jumping height slightly decreased from the general to the specific warm-up and subsequently remained at this significantly lower level, regardless of the condition.

### 3.4 Study III: upper vs. lower body

**FIGURE 3:**
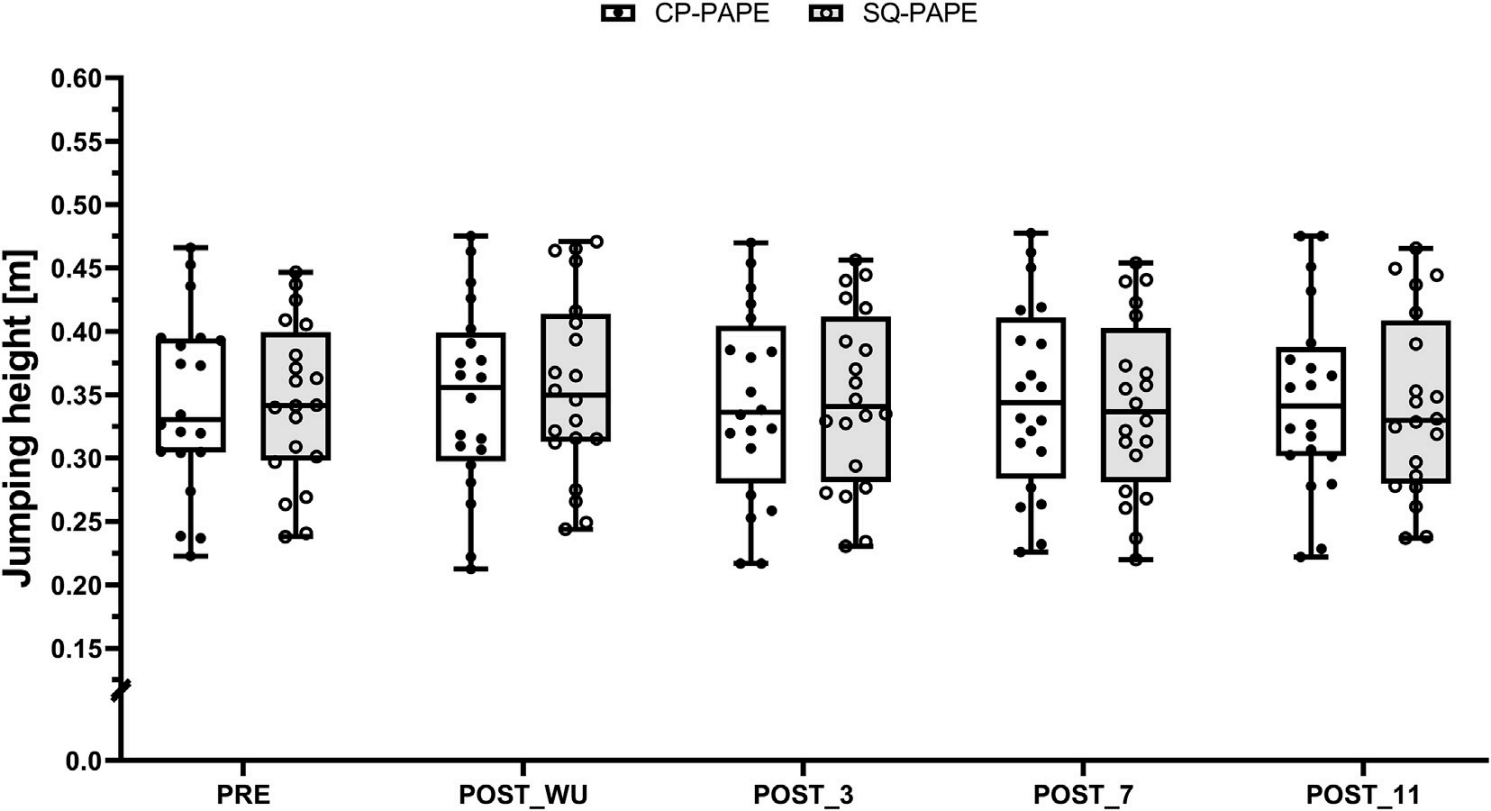
Boxplot (Q1 to Q3, including Median), and Whiskers (showing minimum and maximum values) for Counter-Movement-Jumping height after the general (PRE) and the specific warm-up (POST_WU) as well as 3 (POST_3), 7 (POST_7), and 11 min (POST_11) after the respective PAPE protocols performed at the chest press machine (CP-PAPE; white box) and squat (SQ-PAPE; grey box). Individual values are also depicted (black circles for CP-PAPE and white circles for SQ-PAPE-130).

CMJ did not show a statistically significant interaction effect (F (4, 171) = 1.59, *p* = 0.178, ω_p_^2^ = 0.01 [trivial effect size]), a statistically significant main effect for condition (F (1, 171) = 0.02, *p* = 0.893, ω_p_^2^ < 0.001 [trivial effect size]), or a statistically significant main effect for time (F (1, 171) = 1.80, *p* = 0.131, ω_p_^2^ = 0.02 [small effect size]). Regardless of the condition, pairwise comparison yielded the highest jumps at POST_WU compared to PRE (CP-PAPE: 0.347 ± 0.074 m vs. 0.343 ± 0.071 m, SMD = 0.06 [trivial effect size]; SQ-PAPE: 0.357 ± 0.073 m vs. 0.344 ± 0.063 m, SMD = 0.19 [trivial effect size]), POST_3 (CP-PAPE: 0.347 ± 0.074 m vs. 0.343 ± 0.075 m, SMD = 0.06 [trivial effect size]; SQ-PAPE: 0.357 ± 0.073 m vs. 0.347 ± 0.070 m, SMD = 0.13 [trivial effect size]), POST_7 (CP-PAPE: 0.347 ± 0.074 m vs. 0.347 ± 0.075 m, SMD <0.01 [trivial effect size]; SQ-PAPE: 0.357 ± 0.073 m vs. 0.340 ± 0.069 m, SMD = 0.23 [trivial effect size]), and POST_11 (CP-PAPE: 0.347 ± 0.074 m vs. 0.347 ± 0.073 m, SMD <0.01 [trivial effect size]; SQ-PAPE: 0.357 ± 0.073 m vs. 0.341 ± 0.071 m, SMD = 0.21 [trivial effect size]) (Figure 3).

## 4 Discussion

This series of three separately conducted randomized controlled crossover trials examined how different conditioning protocols affected post-activation performance enhancement (PAPE) in young, healthy, and moderately trained participants. The first study compared submaximal *versus* supramaximal isometric squats, the second examined electrical stimulation as a potential stimulus to induce PAPE, while the third study compared a muscle-specific with an unspecific conditioning activity. In all three studies, our findings did not demonstrate any statistically significant improvement in jump height immediately following the PAPE-conditioning interventions. Notably, there was a significant main effect observed for time, indicating a decrease in jump height post-conditioning across all studies. Nevertheless, this decrement in performance only exhibited trivial effects. Furthermore, our analysis, based on the limits of agreement calculated from our baseline-values, indicated that all observed effects fell within the range of noise, suggesting that the differences observed were not practically meaningful. Thus, although various PAPE conditioning protocols were tested, none showed a significant or relevant enhancement in jump height that might be attributed to PAPE exceeding the warm-up effect.

In our first study, we observed no significant improvements in jumping performance following isometric squatting with 65%1RM or 130%1RM as additional load. Results of similar studies comprising isometric contractions as a conditioning activity exhibit unequivocal results: Miyamoto and colleagues (2011a) reported significant increases in m. triceps surae peak torque during the concentric phase of isokinetic plantar flexion at 180°·s^-1^ immediately following 10 s of maximal voluntary isometric concentric plantar flexion, lasting for another 3 min after the conditioning activity (Miyamoto et al., 2011). Similarly, Rixon and colleagues (Rixon et al., 2007) reported a statistically significant, but trivial (SMD = 0.20) increase in CMJ performance after 3 × 3 seconds of maximal voluntary isometric squats at the smith machine. On the contrary, a series of studies employing various activation protocols, such as a single maximal voluntary isometric squat lasting 3–7 s (Robbins and Docherty, 2005; Pearson and Hussain, 2014; Piper et al., 2020) or 3 x 3–5 s of maximal voluntary isometric contractions during the leg press (Tsolakis et al., 2011) or knee extensor machine (French et al., 2003), either showed no increase or even negative effects on CMJ performance. These studies were however all conducted using maximal voluntary isometric contractions as a preconditioning activity. Employing a first set of a 4-s isometric contraction with a submaximal load of 75%1RM, led to significant improvements in CMJ performance (+2.8%, SMD = 0.34) 4 minutes after preconditioning, but returned to baseline values after adding a second set (Vargas-Molina et al., 2021). Similarly, in a recent study comprising 45 young men with over 6 years of resistance training experience, the experimental group performing 3 × 4 s of isometric squats at 70%1RM exhibited significantly higher CMJ heights (2.7 cm–1.8 cm) 3–9 min after the preconditioning activity compared to the control group (4 min of treadmill running/walking at 6 km/h), whose jump performance remained unchanged (Koźlenia and Domaradzki, 2024). Incorporating an additional third experimental group performing 3 × 5 s of isometric squats at maximal voluntary contraction in a study comprising a similar design (Koźlenia and Domaradzki, 2023), slight increases in jumping height 3 min (for the group performing 3 × 4 s of isometric squats at 70%1RM, SMD = 0.28) and 9 min (for the group performing 3 × 5 seconds of squats at maximal isometric voluntary contraction, SMD = 0.20) after the conditioning activities were reported, respectively. Nevertheless, as all these studies reported only trivial to small positive or negative effects laying within the levels of agreement, we computed based on our baseline values (roughly ±0.05 m). Thus, even though isometric training may exhibit less acute fatigue (Lum and Howatson, 2023) and isometric contractions may induce a post activation potentiation (PAP) effect, lasting up to 5 minutes after brief (≤30 s) maximal voluntary isometric contractions (Pääsuke et al., 2007; Miyamoto et al., 2011; Requena et al., 2011; Pearson and Hussain, 2014; Skurvydas et al., 2019; Gago et al., 2020), it does not seem to be sufficient in inducing PAPE effects when performed as the sole activation condition. Therefore, submaximal isometric contractions may be more effectively utilized as part of a comprehensive warm-up routine incorporating both dynamic movements and isometric contractions.

Similarly, studies on PAPE effects induced via electrical stimulation are scarce and inconclusive. For instance, Dote-Montero and colleagues (Dote-Montero et al., 2021) investigated whether the simultaneous application of whole-body electromyostimulation (5 × 6 s at a stimulation frequency of 100 Hz and a stimulation intensity of 100 mA, pulse duration: 0.2–0.4 milliseconds) led to higher maximal isometric force in the lower extremities than a PAPE protocol consisting of 8 s of maximal isometric split squats alone. Despite both protocols resulting in a significant increase in maximal isometric force, no significant differences between protocols were reported. Additionally, complementary electrical stimulation (stimulation frequency: 100 Hz, maximal tolerable stimulation intensity) of the m. vastus lateralis and medialis during three repetitions of squats with (85%1RM) and without additional load did not alter the 10 m or 30 m sprinting performance in physically trained young men (Sari et al., 2022). Although in our study, participants’ knee extensor muscles were stimulated for a substantially longer duration (total of 90 s) at a lower stimulation frequency of 70 Hz (maximal stimulation intensity: 102 mA) compared to the aforementioned studies, we did not find any PAPE effects on the subsequently performed Counter-Movement-Jump. However, in the acute phase after a comparable session of electrical stimulation, maximal voluntary isometric contraction and power of the knee extensor muscles was reduced by ∼20% accompanied by significant reductions in M-wave suggesting peripherally fatiguing effects (Zory et al., 2005). Thus, our protocol may have induced an excessive stimulation intensity, failing to balance fatigue and potentiation, and thus potentially resulting in fatigue outweighing potentiation (Tillin and Bishop, 2009). This might be further supported by findings of very high creatine kinase concentrations after EMS training in an unfamiliarized sample, indicating severe muscle damage (Kemmler et al., 2015). Overall, electrical stimulation seems not to be superior to traditional methods to induce a PAPE effect. Nevertheless, given the low number of studies investigating possible PAPE effects after an EMS condition protocol, and the complete absence of such studies using the CMJ as an outcome measure, further research exploring different stimulation patterns (e.g., very low stimulation frequencies) is warranted. Moreover, it might be reasonable to disentangle possible cofounding effects of being familiar with the EMS stimulus and training status of the participants.

In our third study, we found no effect of an upper-body conditioning activity (chest press) on subsequently performed Counter-Movement-Jumps. These findings are in contrast with the results of a recently published study of Bartolomei and colleagues (2023), who reported an improvement in Counter-Movement-Jump power 8 min after five repetitions at the bench press with and additional load of 90%1RM, suggesting possible transfer-effects on lower-body performance (Bartolomei et al., 2023). Similarly, another recent study (Caldeira et al., 2023) reported indications of a non-localized PAPE effect, as significant improvements in bench throw heights were observed 4–12 min after 3 × 4 repetitions of clean and jerk (30%1RM to 80%1RM) in male weightlifters. However, again, despite being statistically significant, the size of these effects may be considered trivial (SMD = 0.18) and small (SMD = 0.21–0.30), respectively. Evidence from studies examining a possible transfer effect after unilateral lower extremity exercise to the untrained contralateral leg is also conflicting, with reports of no effects (Power et al., 2021), positive effects (Marín et al., 2014), or even detrimental effects (Andrews et al., 2016) on subsequent performance measures. Nevertheless, overall these changes are within our level of agreements and the realm of what might be expected following unspecific warm-up routines consisting of walking/running, dynamic exercises, and/or stretching (Vetter, 2007; Fradkin et al., 2010). Thus, it is also fair to assume that (muscle-)unspecific PAPE protocols do not exhibit larger improvements compared to general warm-up routines.

Overall, all of our condition activities, even including the traditional PAPE protocols consisting of half-squats, did not yield the expected PAPE effects, thus questioning the additional benefit of PAPE protocols beyond the effect of a comprehensive warm-up routine. A plausible explanation for this discrepancy with previous studies may be attributed to the intensity of our warm-up regimen. While previous studies often implemented a short and low-load aerobic warm-up (e.g., 5 min of cycling at 50% of the participants’ bodyweight (Lowery et al., 2012; Esformes and Bampouras, 2013)), we used a warm-up consisting of cycling at a notably higher, but still moderate resistance (i.e., 150% of the participants’ bodyweight in W) or a combination of cycling and movement specific strength exercises to better reflect what is implemented by athletes in real-life scenarios and traditionally defined as warm-up routine (i.e., consisting of general and sport-specific (e.g., specific stretches and sport-related movements) parts comprising light aerobic activities (e.g., jogging) and resistive exercises (Kulund and Töttössy, 1983; Safran et al., 1989)). Nevertheless, as the PAPE effect is interpreted as an enhancement in a given performance measure from baseline (obtained after the general warm-up) to after the conditioning activity, a more thorough warm-up routine will most likely increase the baseline values, thus reducing the possibility of detecting further improvements induced by the conditioning activity. Depending on the definition, the higher load of our warm-up regimen thus induced a performance effect itself, masking the possible PAPE effect induces by the conditioning activity. This is supported by studies successfully inducing PAPE effects using comparable loads in their preconditioning activities (Suchomel et al., 2016; Kobal et al., 2019; Caldeira et al., 2023). Therefore, to further disentangle possible PAPE from warm-up effects, future research should (i) focus on providing a clear definition to distinguish between PAPE and (specific) warm-up routines and (ii) provide an adequate warm-up prior to conducting baseline measurements, thus reducing the risk of masking potential PAPE effects with warm-up effects.

A limitation of our studies is the fixed time points and given loads for all athletes, which do not allow for finding the individually highest PAPE effect (McCann and Flanagan, 2010). This is especially relevant, as a training block of heavy strength training may lead to PAPE effects being more pronounced and visible in closer temporal proximity to the conditioning activity (Miyamoto et al., 2013), thus increasing the heterogeneity in a sample. However, to counteract these problems, we aimed at recruiting a homogenous group of participants, possessing at least 2 years of experience, and regularly engaging in strength training. Moreover, we tested the Counter-Movement-Jump at several time points, which meta-analyses described as optimal to ensure finding the highest post-activation improvements (Gouvêa et al., 2013; Lesinski et al., 2013; Wilson et al., 2013; Seitz and Haff, 2016; Garbisu-Hualde and Santos-Concejero, 2021). Nevertheless, compared to part of previously published research our participants exhibited a lower 1RM related to their bodyweight. Given that athletes of a more advanced training status may exhibit larger PAPE effects, it is possible that including athletes of a more advanced training status would potentially have led to PAPE effects.

In conclusion, despite employing different conditioning activities ranging from traditional PAPE protocols comprising half-squats or isometric squats to different activation protocols such as electrical stimulation, jump performance post-conditioning did not further increase compared to post-warm-up. Thus, a comprehensive warm-up routine incorporating dynamic, movement-specific exercises, and isometric contractions with moderate to high additional loads might be most effective as a pre-exercise routine.

## Data Availability

The datasets presented in this study can be found in online repositories. The names of the repository/repositories and accession number(s) can be found below: https://osf.io/hj9ux/.
